# Focal Prostate Stereotactic Body Radiation Therapy With Correlative Pathological and Radiographic-Based Treatment Planning

**DOI:** 10.3389/fonc.2021.744130

**Published:** 2021-09-15

**Authors:** Elisha Fredman, Bryan Traughber, Michael Kharouta, Tarun Podder, Simon Lo, Lee Ponsky, Gregory MacLennan, Raj Paspulati, Bradley Ellis, Mitchell Machtay, Rodney Ellis

**Affiliations:** ^1^Department of Radiation Oncology, Seidman Cancer Center, University Hospitals, Cleveland Medical Center, Cleveland, OH, United States; ^2^Department of Radiation Oncology, Penn State University, Milton Hershey Medical Center, Hershey, PA, United States; ^3^Department of Radiation Oncology, University of Washington School of Medicine, Seattle, WA, United States; ^4^Department of Urology, Seidman Cancer Center, University Hospitals, Cleveland Medical Center, Cleveland, OH, United States; ^5^Department of Pathology, University Hospitals, Cleveland Medical Center, Cleveland, OH, United States; ^6^Department of Radiology, University Hospitals, Cleveland Medical Center, Cleveland, OH, United States

**Keywords:** prostate, radiation, SBRT, MRI, focal

## Abstract

**Introduction:**

Advances in multiparametric MRI (mpMRI) combining anatomic and functional imaging can accurately identify foci of adenocarcinoma within the prostate, offering the possibility of partial gland therapy. We performed tandem prospective pilot trials to investigate the feasibility of focal prostate SBRT (f-SBRT) based on correlating diagnostic mpMRI and biopsies with confirmatory pathology in treatment planning.

**Materials and Methods:**

Patients with pathologic focal Gleason 6–7 disease and a corresponding PIRADS 4–5 lesion on mpMRI underwent targeted and comprehensive biopsies using MRI/ultrasound fusion under electromagnetic sensor navigation. After rigorous analysis for imaging biopsy concordance, five of 18 patients were eligible to proceed to f-SBRT. Chi-squared test was used for differences from expected outcomes, and concordance was estimated with binomial distribution theory and Wilson’s method.

**Results:**

Six patients had Gleason 6 and 12 had Gleason 3 + 4 disease (mean PSA: 5.8 ng/ml, range: 2.2–8.4). Absolute concordance was 43.8% (95% CI: 0.20, 0.64). Patterns of discordance included additional sites of ipsilateral disease, bilateral disease, and negative target. Five were upstaged to a new NCCN risk category necessitating treatment escalation. The five patients with concordant pathology completed three-fraction f-SBRT with sparing of the surrounding normal structures (including contralateral neurovascular bundle), with no reported grade 2+ toxicities and favorable PSA responses (mean: 41% decrease).

**Conclusions:**

On our pilot trials of f-SBRT planning using rigorous imaging and pathology concordance, image-guided confirmatory biopsies frequently revealed additional disease, suggesting the need for caution in partial-gland therapy. For truly focal disease, f-SBRT provided excellent dosimetry, minimal toxicity, and encouraging biochemical response. **Clinical Trial Registration**: www.clinicaltrials.gov, NCT02681614; NCT02163317.

## Introduction

Prostate cancer represents the sole malignancy where the entire organ is standardly targeted. There is a growing concern over the risk of overtreating patients who may not be at risk of dying from the disease. Advances in multiparametric MRI (mpMRI) and prostate-specific positron emission tomography (PET), combining anatomic and functional imaging, can more accurately identify high-grade and poorly differentiated foci within the prostate, characterizing their aggressiveness and malignant potential (1–5). As a result, there has arisen the possibility of partial gland therapy and image-guided focal dose escalation, with the goal of further sparing the normal tissues to reduce treatment-related toxicity (6–12).

A major limitation of mpMRI-directed partial gland therapy is the variable concordance between imaging and histopathology (13–15). Rates in the literature range from 12.1% to 69.7% when assessing the accuracy of mpMRI based on confirmatory biopsies and vary depending on Prostate Imaging Reporting and Data System (PIRADS) risk (4, 5, 13, 16). Characterizing the ability of mpMRI to truly reveal the extent of the disease in the prostate is underway, including as an exploratory endpoint in NRG GU-005, yet it is still not completely understood. Furthermore, there has been little reported on the resulting therapeutic implications of histopathologic correlation with imaging findings, and as such, the viability of delivering partial prostate treatments based on imaging-defined targets.

In this context, we performed tandem prospective pilot studies to better elucidate these diagnostic and interventional challenges in the context of planning partial gland therapy. In an initial cohort, we examined the concordance of diagnostic sextant biopsy and mpMRI with image-guided targeted and comprehensive biopsies. In a second cohort, those found to have complete pathologic concordance of radiographically localized disease received focal prostate stereotactic body radiation therapy (f-SBRT) utilizing a novel methodology for target volume delineation. As such, we determined the resulting clinical and radiotherapeutic implications of these radiographic and histopathologic findings.

## Materials and Methods

The Institutional Review Board approved two prospective pilot trials of targeted prostate biopsies and f-SBRT. The initial protocol included six patients with a single focus of disease measuring ≥5 mm based on diagnostic 3-Tesla (3T) mpMRI. Eligibility criteria included low- and intermediate-risk adenocarcinoma of the prostate, clinical stage T1c-T2a, Gleason score (GS) ≤7 (dominant pattern: 3), PSA ≤15 ng/ml, Eastern Oncology Cooperative Group (ECOG) performance status 0–1, and the ability to undergo MRI (**Table 1**). Stage group was defined as per the NCCN guidelines, version 3.2018 (17).

**Table 1:** Descriptive patient characteristics.

Trial	No.	Age	T stage	PSA	Gleason score	Stage/Grade group	Risk	Lesion No.	SHIM	IPSS
**Cohort 1**	1	65	1c	6.9	3 + 4 = 7	IIB/2	Intermediate	2	18	6
	2	50	1c	6.4	3 + 4 = 7	IIB/2	Intermediate	1	1	10
	3	79	1c	4.6	3 + 4 = 7	IIB/2	Intermediate	2	1	11
	4	72	1c	8.4	3 + 4 = 7	IIB/2	Intermediate	1	25	6
	5	65	1c	5.6	3 + 4 = 7	IIB/2	Intermediate	1	2	19
	6	60	1c	8.4	3 + 4 = 7	IIB/2	Intermediate	2	5	19
**Cohort 2**	1	53	1c	5.2	3 + 4 = 7	IIB/2	Intermediate	1	25	2
	2	66	1c	4.9	3 + 4 = 7	IIB/2	Intermediate	1	21	7
	3	64	1c	3.9	3 + 3 = 6	I/1	Very low	1	21	18
	4	73	1c	2.2	3 + 3 = 6	I/1	Very low	1	19	3
	5	59	1c	5.9	3 + 4 = 7	IIB/2	Intermediate	1	25	1
	6	76	1c	6.6	3 + 3 = 6	I/1	Low	2	10	4
	7	64	1c	7.5	3 + 3 = 6	I/1	Low	2	11	12
	8	54	1c	6.7	3 + 3 = 6	I/1	Very low	1	12	10
	9	75	1c	4.5	3 + 3 = 6	I/1	Very low	1	1	7
	10	76	1c	5.9	3 + 4 = 7	IIB/2	Intermediate	1	1	18
	11	70	1c	4.3	3 + 4 = 7	IIB/2	Intermediate	1	17	11
	12	71	1c	7.2	3 + 4 = 7	IIB/2	Intermediate	2	5	8

SHIM, sexual health inventory for men; IPSS, International Prostate Symptom Score.

The subsequent protocol comprises 12 patients and was designed to evaluate the therapeutic implications of histopathological correlation with mpMRI through the feasibility of targeted f-SBRT of the solitary lesion. If whole-gland and targeted prostate biopsies confirmed a single focus of cancer with no other sites of disease in >5% of any core, the patient was eligible for f-SBRT.

All subjects were initially diagnosed based on an elevated screening PSA and underwent a standard trans-rectal ultrasound (TRUS)-guided 12-core sextant biopsy for pathologic diagnosis, followed by staging pelvic 3T mpMRI with body coil (T2, T1 with contrast, DCE, and DWI sequences). If the biopsy results correlated exclusively with a PIRADS 4–5 lesion on mpMRI measuring ≥5 mm and without evidence of extraprostatic extension, the patient could be eligible to enroll on trial (**Table 1**), and consent was obtained.

**Figure 1:**
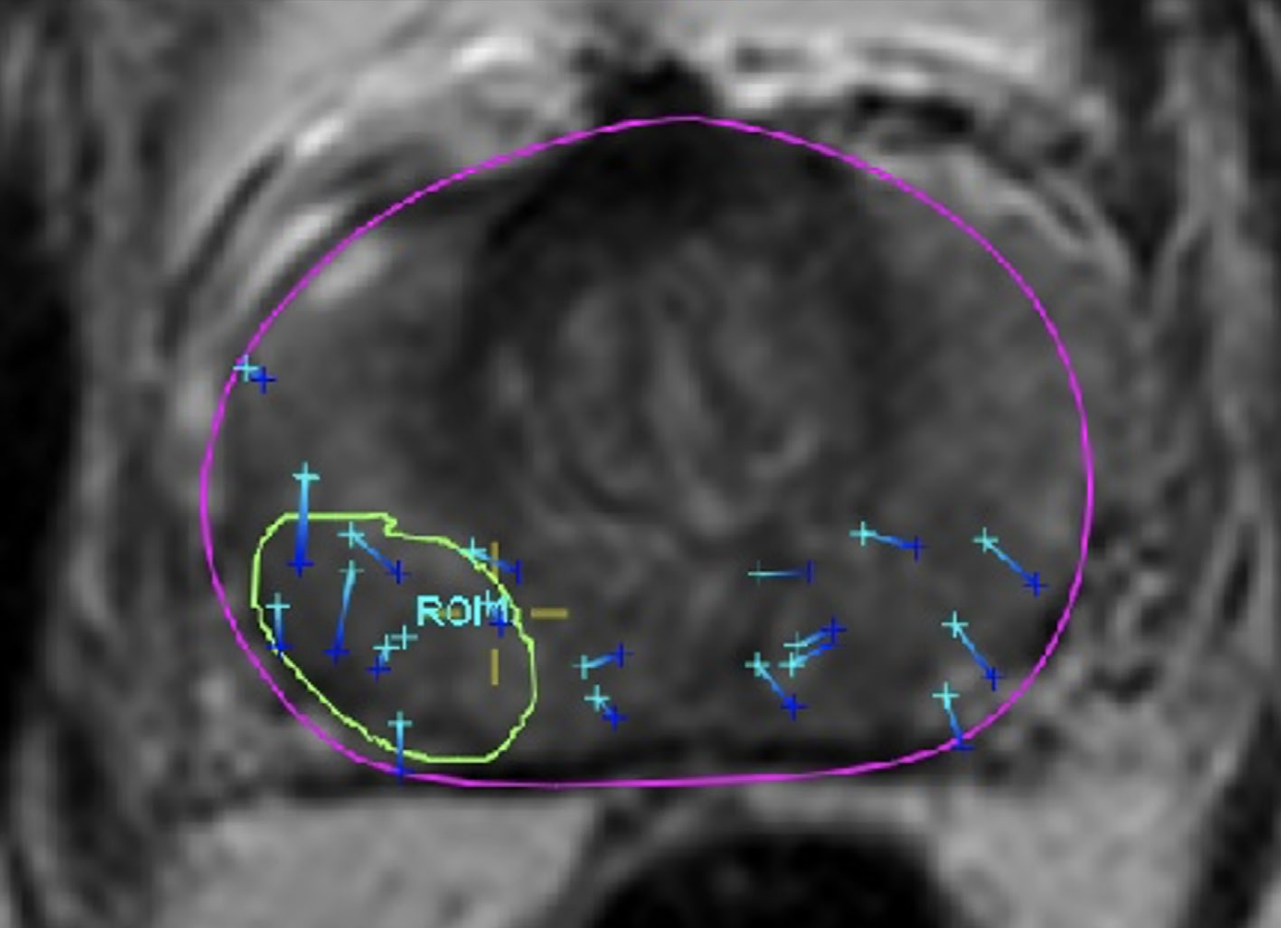
Targeted and repeat whole-gland biopsies using the UroNav needle guidance system.

Region-of-interest (ROI) delineation was performed using the FDA-approved DynaCAD digital imaging system, and targeted biopsies were obtained using UroNav transperineal needle tracking (**Figure 1**). Gold fiducials were placed in the biopsy cavity to track each targeted core as part of a novel CHAMPS^®^ methodology (Correlated Histopathology and Marker Placement System), applying both functional and anatomic data to planning target volume (PTV) determination. After the target was sampled, a standard 12-core biopsy was repeated to confirm no additional disease. On the therapeutic protocol, subjects with complete radiographic/histopathologic correlation began f-SBRT treatment planning. Discordance was defined as: (i) pathologically proven disease outside of the PIRADS 4–5 MRI lesion, with the exception of a core with ≤5% GS 6; (ii) upstaging in risk group to dominant pattern 4 disease or higher; and (iii) all-negative biopsies of the index lesion.

CT simulation with and without contrast and 4D-CT were performed with custom immobilization. A rectal balloon was placed to enhance positional reproducibility, and an integrated plastic scintillating device (OARtrac Angiodynamics, Latham, NY) recorded the *in vivo* dose at the anterior rectal wall interface. Placement of a rectal spacer hydrogel, previously shown to decrease treatment-related rectal toxicity in whole gland radiotherapy, was at the discretion of the treating physician (18). The gross tumor volumes (GTV) on mpMRI and planning CT scans were combined to generate an internal target volume (ITV). Marked sites of biopsy-proven disease were added to create the clinical target volume (CTV), expanded by a uniform 5 mm for a final PTV. Inverse treatment planning was performed using volumetric modulated arc therapy (VMAT).

Three fractions of f-SBRT were delivered every other day to a total dose of 29.25 Gy (9.75 Gy per fraction) to obtain a BED of >268 Gy, with an assumed α/β for prostate cancer of 1.2 (19). Other published experiences of HDR prostate brachytherapy have attained this dose prescribing 30 Gy in three fractions (20). In order to account for intrafractional target motion, four CBCT scans were obtained, the first prior to treatment, and after delivery of each 3.25 Gy. This additional 12 cGy combined to a total of approximately 10 Gy per fraction. OAR dose constraints were derived from NRG Oncology RTOG 0938 (21).

Patients were assessed for treatment-related toxicities, using the Common Terminology Criteria for Adverse Events (CTCAE) survey, and PSA changes every 3 months for 24 months. Baseline pretreatment sexual function and urinary symptom scores were established using the Sexual Health Inventory for Men (SHIM) and International Prostate Symptom Score (IPSS) tools and were monitored for changes every 3 months following radiation. Patients underwent a midtreatment MRI, and surveillance MRI was performed at 6, 12, and 24 months. Treatment failure was determined either by the Phoenix definition or a positive biopsy.

Descriptive statistics were used to report outcomes of concordance and acute and delayed toxicity from prostate f-SBRT. Chi-squared test was used to determine differences between expected and observed frequencies of concordance. Concordance rate and its 95% confidence interval (CI) were estimated based on binomial distribution theory and Wilson’s method (22). As a primarily proof-of-principle study, it was not designed to have power for detecting significant difference in clinic outcomes (i.e., toxicities, survival, or QOL).

## Results

Patient characteristics were balanced across the two trials (**Table 1**). All patients had stage T1c disease: 12 were GS 3 + 4 = 7 and six were GS 6. Five of the six patients in the initial cohort were treated with LDR brachytherapy while one underwent external beam radiation (EBRT) due to a high International Prostate Symptom Score (IPSS). One patient in the second cohort declined targeted biopsy and proceeded to radical prostatectomy.

Among the 16 patients who completed repeat confirmatory biopsies, seven were pathologically concordant with diagnostic pathology and imaging (2/5 in cohort 1, 5/11 in cohort 2) (**Table 2**). The concordance rate (agreement rate) was 43.8% (95% CI: 0.20, 0.64, respectively). For the nine patients with discordant biopsy results, five were up-staged, three remained the same, and one was down-staged.

**Table 2:** Pathology and mpMRI concordance.

Subject	Sextant biopsy	mpMRI	Targeted biopsy	Concordance	Explanation	Therapeutic implication
**1**	**7(3 + 4)-L-Ant/Mid6-R-Ant/Mid**	**L-Ant Tz**	**7(3 + 4)-TargetRight negative**	**Concordant**	**Concordant**	**LDR-BT**
**2**	**7(3 + 4)-Right**	**R-Ant Tz**	**6-Target**	**Concordant**	**Concordant**	**LDR-BT**
3	7(3 + 4)-L-Ant/Mid6-R-Mid	L-BasR-Mid	7(3 + 4)-L-Ant, 6-L-Mid6-R-Cen/Mid/Ant	Discordant	Additional high volume i/l GS 6	LDR-BT
4	7(3 + 4)-L-Ant	L-Ant/Mid	6-R-Bas	Discordant	Target negative	LDR-BT
5	7(3 + 4)-Left	L-Pos/Pz	NA	NA	NA	IPSS elevated, ADT
6	7(3 + 4)-Left	L-Lat Pz	7,8,9-b/l disease	Discordant	Diffuse b/l high grade tumor	LDR-BT
**1**	**7(3 + 4)-R-LM**	**R-Lat Pz**	**6-Target**	**Concordant**	**Concordant**	**Focal SBRT**
**2**	**7(3 + 4)-R-LA**	**R-Ape Pz**	**7(3 + 4)-Target**	**Concordant**	**Concordant**	**Focal SBRT**
**3**	**7(3 + 4)-R-Ape**	**R-Ape**	**7(3 + 4)-Target**	**Concordant**	**Concordant**	**Focal SBRT**
**4**	**7(3 + 4)-L-LB**	**L-LB**	**7(3 + 4)-Target**	**Concordant**	**Concordant**	**Focal SBRT**
**5**	**7(3 + 4)-L-LB/L-MB**	**LB**	**7(3 + 4)-Target**	**Concordant**	**Concordant**	**Focal SBRT**
6	6-L-Mid	L-Pos Pz	6-Target6-R-Bas/Mid/Lat	Discordant	C/l GS 6	Whole gland SBRT
7	6-L-LM	L-Lat Pz	Target negative6-L-Ant	Discordant	Target negative, additional i/l GS 6	Active surveillance
8	6-R-Mid	R-Mid Tz	7(4 + 3)-Target7(3 + 4)-R-LA	Discordant	Additional i/l GS 7	VMAT, ADT
9	6-L-LM/L-LB	L-Ape/Mid	7(3 + 4)-Target7(4 + 3)-R-Mid	Discordant	C/l GS 7	Proton RT
10	6-L-Ant/Mid	L-Ant Pz/Tz	8(3 + 5)-Target	Discordant	High-volume GS 8	Surgery
11	6-Right Tz	Right Tz; B/l Pz	NA	NA	NA	Surgery
12	6-R-Bas/Ape	R-Ape Tz	7(3 + 4)-Target6-R-Ant (50%)	Discordant	Additional high-volume GS 6	Whole gland SBRT

L-Ant, left anterior; L-Lat, left lateral; L-Mid, left mid; L-Ape, left apex; L-LM, left lateral mid; L-Bas, left base; L-Pos, left posterior; L-LB, left lateral base; L-MB, left medial base; R-Cen, right center; R-Lat, right lateral; R-Mid, right mid; R-Ape, right apex; R-LM, right lateral mid; R-Bas, right base; R-LA, right lateral apex; Pz, peripheral zone; Tz, transitional zone; LDR-BT, low-dose rate brachytherapy; GS, Gleason score; i/l, ipsilateral; c/l, contralateral; b/l, bilateral.

NA, Not-applicable.

Bold font is emphasizing the subjects in whom concordance was found between pathology and imaging.

In the first cohort, discordance was due to additional ipsilateral high-volume GS 6 disease, negative targeted biopsy, and bilateral high-grade disease. This final patient was upstaged from intermediate- to high-risk status. All underwent definitive I-125-based LDR brachytherapy. In the second cohort, discordance was due to additional ipsilateral multifocal disease in three patients, contralateral cancer in two, and GS 8 disease within a presumed GS 6 target lesion in one (**Table 2**). Four patients were upstaged to higher-risk groups. Discordant patients completed whole-gland SBRT (2), fractioned EBRT with photons (1) and protons (1), prostatectomy (1), and active surveillance (1). One received 6 months of ADT. Patients in the second cohort who demonstrated radiographic and pathologic concordance successfully completed f-SBRT as described below.

**Figure 2:**
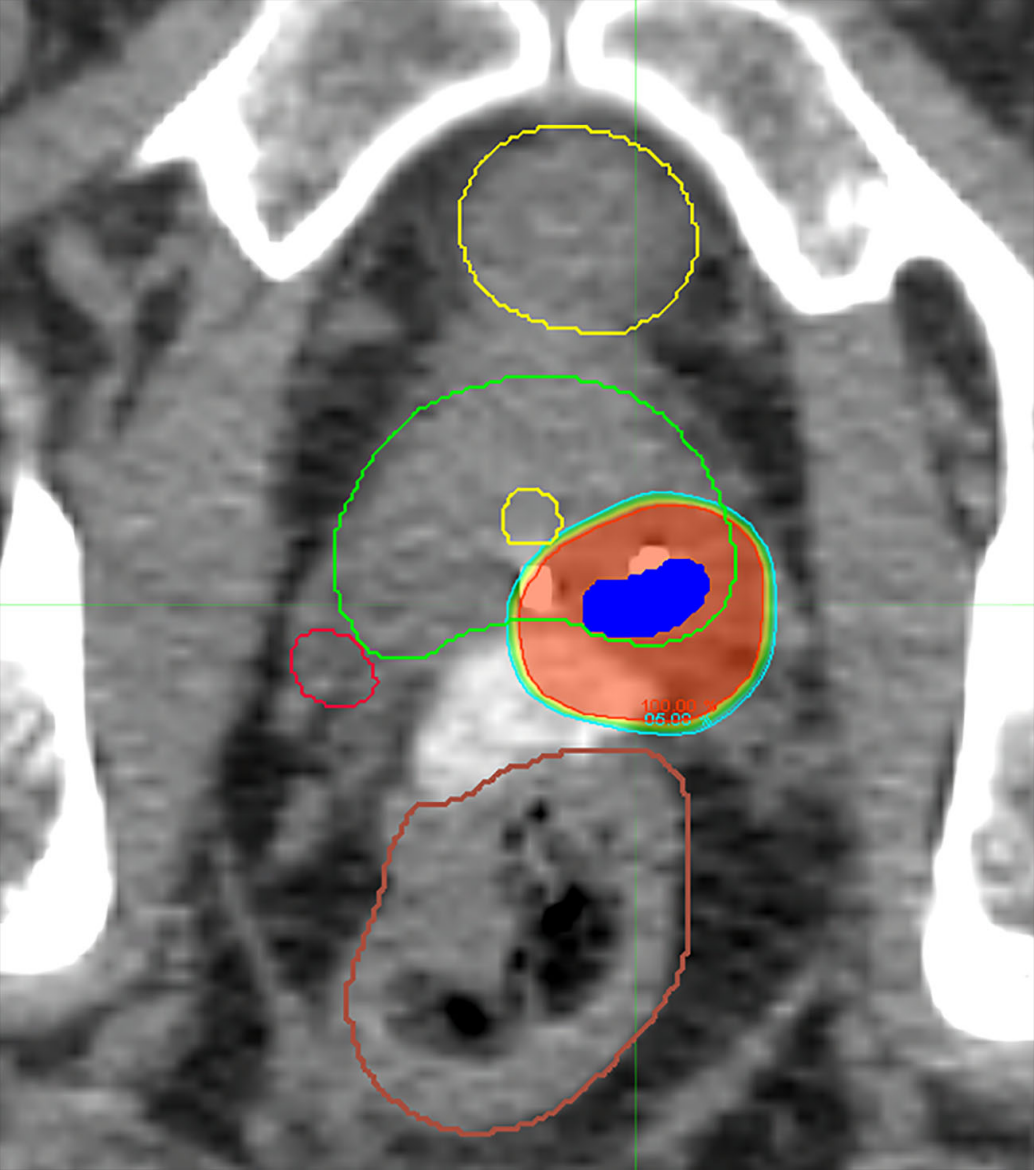
Representative axial CT slice with isodose lines from a delivered f-SBRT plan demonstrating sparing of the uninvolved prostate gland, bladder, rectum, urethra, and contralateral neurovascular bundle.

Two of the five treated subjects had biopsy-proven disease extending beyond the 5-mm ITV expansion which was incorporated into the final target volume, thus validating the concept for the CHAMPS^®^ methodology. Appropriate target volume coverage (PTV D_90_ = 100%, PTV D_95_ > 95%) was achieved (mean PTV D_90_ = 100%; mean PTV D_95_ = 98%) (**Figure 2**, **Table 3A**). Limited heterogeneity was demonstrated with acceptable maximum and minimum doses to 0.03 cm^3^ of the PTV (mean PTVmax = 109%; mean PTVmin = 96%). Conformality was characterized by normal tissue V105 of 0.78 cm^3^ (goal <5 cm^3^), mean conformality index of 1.14, and ratio of the 50% isodose line to PTV (Ratio_50%_) of 6.34. A learning curve was apparent within this novel treatment paradigm, and target dosimetry improved with subsequent f-SBRT plans.

**Table 3:** f-SBRT dosimetry; Gy (% of prescription dose).

(a) Target volume dosimetry												
Patient	PTV D95		PTV D90		PTV max	PTV min	Norm. tissue V105			C.I.	Ratio_50%_	
**1**	97.6%		100%		34.22 (117)	27.90 (95)	3.9 cm^3^			1.26	8.9	
**2**	95.6%		100%		31.24 (107)	28.20 (96)	0 cm^3^			1.08	5.3	
**3**	100%		100%		30.97 (106)	28.58 (98)	0 cm^3^			1.20	6.6	
**4**	98.2%		100%		31.25 (107)	27.88 (95)	0 cm^3^			1.07	5.08	
**5**	98%		100%		31.29 (107)	27.87 (95)	0 cm^3^			1.1	5.8	
**(b) Organ at risk dosimetry**												
**Organ**		**Volume**		**Parameter**	**Patient #1**	**Patient #2**	**Patient #3**	**Patient #4**	**Patient #5**		**Mean**	**NRG GU 005 parameter**
**Rectum**		Max		≤30.71 (105)	23.15 (79)	29.56 (101)	10.39 (36)	30.06 (103)	25.95 (89)		23.82 (81)	≤38.06 (105)
		D10		≤26.33 (90)	10.05 (34)	15.90 (54)	7.46 (26)	10.74 (37)	5.96 (20)		10.02 (34)	≤32.63 (90)
		D20		≤23.40 (80)	5.51 (19)	12.08 (41)	6.20 (21)	8.11 (28)	4.07 (14)		7.19 (25)	≤29.00 (80)
		D50		≤14.63 (50)	1.59 (5)	2.62 (9)	0.58 (2)	4.37 (15)	0.83 (3)		2.00 (7)	≤18.13 (50)
**Bladder**		Max		≤30.71 (105)	11.29 (39)	2.37 (8)	1.73 (6)	28.91 (99)	27.70 (95)		14.40 (49)	≤38.06 (105)
		D10		≤26.33 (90)	1.43 (5)	0.92 (3)	0.57 (2)	8.06 (28)	4.64 (16)		3.12 (11)	≤18.12 (50)
		D50		≤14.63 (50)	0.39 (1)	0.57 (2)	0.27 (1)	2.57 (9)	0.33 (1)		0.83 (3)	NA
**PB**		Max		≤29.25 (100)	1.10 (4)	7.75 (26)	2.23 (8)	0.63 (2)	0.60 (2)		2.46 (8)	≤36.25 (100)
		D3cc		≤15.80 (54)	0.75 (3)	0.0 (0)	0.65 (2)	0.31 (1)	0.41 (1)		0.42 (1)	≤19.90 (55)
		Mean		NA	0.68 (2)	2.88 (10)	1.07 (4)	0.41 (1)	0.48 (2)		1.10 (4)	NA
**Urethra**		Max		≤31.30 (107)	28.80 (98)	19.43 (66)	30.75 (105)	14.33 (49)	26.78 (92)		26.78 (92)	≤38.78 (107)
**NVBc**		Max		NA	9.96 (34)	17 (58)	10.59 (36)	11.84 (40)	13.18 (45)		12.51 (43)	NA
		Mean		NA	7.27 (25)	8.36 (29)	3.59 (12)	9.27 (32)	11.29 (39)		7.96 (27)	NA

Max, maximal dose to 0.03 cm^3^; PTV, planning target volume; C.I., Conformality index; PB, penile bulb; NVBc, contralateral neurovascular bundle.

NA, Not-applicable.

Additionally, favorable dosimetry for normal tissue avoidance was achieved for all of the f-SBRT treatment plans, meeting parameters far below dose constraints set based on the current NRG trial for prostate SBRT, GU005 (**Table 3B**). Mean values for maximum rectal dose, D10, D20, and D50 were 23.82, 10.02, 7.19, and 2.00 Gy, respectively. Similarly, mean values for bladder constraints, including maximum dose, D10, and D50 were 14.4, 3.12, and 0.83 Gy, respectively. Mean penile bulb maximum dose and mean dose were 2.46 and 1.10 Gy, respectively, and mean urethral maximum dose was 26.78 Gy. Finally, relative sparing of the contralateral neurovascular bundle was achieved, with average maximum and mean dose of 12.51 and 7.96 Gy, respectively.

As primarily a phase-I pilot study, clinical outcomes are limited, but preliminary biochemical response and symptom scores are presented. All evaluable patients had a decrease in PSA on first posttreatment measurement (mean: 46.8%). At a mean follow-up of 30 months (range: 6–57), no biochemical failures have occurred. With regard to urinary function, all patients experienced stable or improved lower urinary tract symptoms on the 3-month posttreatment self-reported IPSS tool, with a mean decrease of 1.4 points. Sexual function measured at 3 months was preserved with only one patient reporting a lower SHIM score; the other four patients with either stable or slightly improved reported function, for a mean decrease of 0.6 points (**Table 4**).

**Table 4:** Pretreatment and 3-month posttreatment IPSS and SHIM scores.

Patient	IPSS			SHIM		
	Pre-SBRT	Post-SBRT	Change	Pre-SBRT	Post-SBRT	Change
**1**	2	2	0	25	25	0
**2**	7	8	+1	21	16	−5
**3**	18	17	−1	1	1	0
**4**	11	7	−4	17	19	+2
**5**	8	5	−3	5	5	0
**Mean**	9.2	7.8	−1.4	13.8	13.2	−0.6

SHIM, sexual health inventory for men; IPSS, International Prostate Symptom Score.

## Discussion

While early experiences and feasibility have been published investigating partial organ SBRT for genitourinary cancer of the kidney (23, 24), SBRT for partial prostate treatment has yet to be well explored. Focal therapy for the treatment of prostate cancer is a potentially promising modality that may offer advantages in treatment-related toxicity, yet questions remain regarding patient selection, accuracy of diagnostic methods, and localization techniques for treatment delivery. On our prospective tandem studies, we found only a 43.8% rate of agreement (seven of 16 patients). Five patients were upstaged to higher-risk groups, indicating more involved definitive therapy. Even with the application of modern imaging and the novel DynaCAD system for MRI-fusion biopsies, our concordance results are consistent with previously published reports (13–15) and suggest the need for sincere equipoise in delivering partial prostate therapy.

To the best of our knowledge, this is the first prospective series analyzing the therapeutic implications of combined mpMRI with confirmatory biopsies for focal prostate SBRT. Previous studies have shown inconsistent concordance even within the context of whole-gland biopsies. A meta-analysis of 16 studies totaling 1,926 men showed that MRI- and TRUS-guided prostate biopsies had a 15% and 19% false-negative rate, respectively (25). A second meta-analysis comparing targeted with whole-gland biopsies found no clear benefit of one over the other (26). Diagnostic capabilities have improved with advances in imaging (27–31), though with imperfect results, and within the context of general diagnostic fidelity prior to whole-gland therapy. Our trials uniquely assessing the ability to identify exclusively focal disease support a multilayered diagnostic process for geographically accurate disease detection. This work expands on previous published work using advanced image-guided focal dose escalation using brachytherapy (32). Three independent platforms were integrated for maximal biopsy precision and with this comprehensive approach, we both uncovered clinically relevant findings and preemptively mapped the regions of disease to better guide stereotactic radiotherapy.

Experiences with other targeted prostate therapy modalities have been reported in the literature (8). Of concern, however, is the reliance primarily on MRI alone to identify significant disease (33). The most robust reports of biochemical progression-free survival are in the context of brachytherapy, with rates of 91.5% and 78.1% at 5 and 8 years, respectively (34). The application of SBRT for focal therapy is an innovative approach, not yet routinely included among reviews of focal ablative techniques (35). Advantages of SBRT include treatment *via* an existing linear accelerator as well as the capability to perform precision pretreatment and intrafractional dosimetric assessments.

On the interventional trial, five of 11 patients were treated with f-SBRT (**Figure 2**). PTV coverage was excellent, with limited heterogeneity and appropriate sparing of the surrounding normal tissue (**Table 3A, B**). Even with the variability of lesion location relative to the urethra, good sparing was achieved in all three plans. Doses to all OARs were substantially less than the limits on the current NRG trial, including relative sparing of the contralateral neurovascular bundle (**Table 3B**). At 3 months following f-SBRT, patient self-reported urinary and sexual function were preserved (**Table 4**).

Data is scarce regarding the quantitative advantages of definitive partial prostate SBRT *vs*. whole-gland therapy. Amini et al. demonstrated theoretical feasibility of delivering hemigland-sparing radiation with predicted dosimetric improvements (36). Kishan et al. designed five-fraction comparison plans and quantified the expected dosimetric advantage (7). They specifically noted favoring a “focused” hemigland *vs*. “ultrafocal” targeted which did not necessitate precise image fusion and rigorous radiological-pathological correlation. Our goal in developing the described diagnostic and therapeutic methodology was specifically to this end. A detailed dosimetric comparison of focal *vs*. whole-gland SBRT for the three treated patients is beyond the scope of this work. Prostate SBRT in general and focal approaches such as f-SBRT specifically may be a way to help limit patient morbidity and deserves further investigation.

There are several methodological and technological limitations to our study. Communication between the surface EM beacon and ultrasound receiver unit of the UroNav system is associated with inherent uncertainty. We accounted for this by obtaining five targeted biopsies to achieve comprehensive lesion assessment. Furthermore, both components require commissioning which is subject to human inaccuracies. Commissioning was performed by a medical physicist with expertise in machine-assisted prostate brachytherapy to minimize this potential confounding. Deformable TRUS/MRI registration also contains uncertainty associated with rectal deformity. Novel methodologies for targeted biopsy and focal treatment require technical expertise and a steep learning curve was apparent. Finally, our studies have the inherent limitations of being nonrandomized pilot trials from which definitive conclusions cannot be drawn.

## Conclusions

In conclusion, on our tandem prospective pilot trials of a novel methodology for radiographic and pathologic correlation of focal prostate cancer in preparation for f-SBRT, confirmatory biopsies revealed additional disease in the majority of subjects. A portion was upstaged with therapeutic implications. Responsible equipoise must be taken when planning partial prostate therapy based on MRI sequencing and would benefit from precise histopathologic correlation. For truly focal disease, three-fraction f-SBRT was successfully delivered to a comprehensive target with advantageous dosimetry and favorable early patient outcomes.

## Data Availability Statement

The raw data supporting the conclusions of this article will be made available by the authors, without undue reservation.

## Ethics Statement

The studies involving human participants were reviewed and approved by University Hospitals Seidman Cancer Center Internal Review Board. The patients/participants provided their written informed consent to participate in this study.

## Author Contributions

EF and BT were co-PIs on the trial, enrolled patients on study, and contributed to the writing and revision of the manuscript. EF wrote the first and final drafts of the manuscript. RE was the first PI and primary developer of the clinical trial and enrolled patients to the study. TP was vital in clinical trial design, development of methodology, and editing of the manuscript. SL and MM contributed to trial design and development. LP assisted with trial design and performed many of the prostate biopsies on trial. GM was the primary pathologist, and RP was the primary radiologist for the trial. All authors contributed to the article and approved the submitted version.

## Funding

This work was supported by Elekta, Stockholm, Sweden (monetary funding) and Philips, Andover, MA (provided the UroNav system for use) (grant numbers 16053.01.N0442 and 15976.01.L2397). The funders were not involved in the study design, collection, analysis, interpretation of data, the writing of this article or the decision to submit it for publication. Of the authors above, those directly impacted by these grants are RE, BT, and MM. There was also support from the Case Comprehensive Cancer Center Core grant (grant number 2P30CA043703-28). Part of this manuscript was presented as an abstract at the American Society for Radiation Oncology Annual Meeting, San Antonia TX, October 2018, and further findings were presented at the American Radium Society Annual Meeting, Dana Point CA, April 2019.

## Conflict of Interest

BT reports grants from Philips Healthcare and from Elekta during the conduct of the study. MM reports nonfinancial support from Elekta and personal fees and nonfinancial support from Philips during the conduct of the study. RE reports nonfinancial support from Elekta and personal fees and nonfinancial support from Philips during the conduct of the study. In addition, RE has two patents U.S. Patents 7831293 and 10842469 with royalties paid by Philips. SL reports past travel and research support from Elekta when the study was developed and current membership of the Elekta ICON Gamma Knife Expert Group.

The remaining authors declare that the research was conducted in the absence of any commercial or financial relationships that could be construed as a potential conflict of interest.

## Publisher’s Note

All claims expressed in this article are solely those of the authors and do not necessarily represent those of their affiliated organizations, or those of the publisher, the editors and the reviewers. Any product that may be evaluated in this article, or claim that may be made by its manufacturer, is not guaranteed or endorsed by the publisher.
